# Milk Sickness, Poison, Tires, &c.

**Published:** 1856-01

**Authors:** W. H. Byford

**Affiliations:** Evansville, Indiana


### SELECTIONS.

---

*Milk Sickness, Poison, Tires, &c.* By W. H. Byford, M. D., of Evansville, Indiana.

All the above synonyms are used to designate a well-marked and distinct disease which prevails over a large district of the U. S. A., but which is ignored by every systematic writer on medicine that I have ever examined. Its very existence is denied by many eminent and influential men in the profession. These circumstances serve to exclude it from the approved works on the practice of medicine. Hence all young practitioners are deprived of the proper source of information on the subject. These considerations, I think, justify journalists in occasionally admitting to their pages even rigidly systematic essays upon it, for the benefit of the junior members of the profession, provided that such is the result of the experience of the authors. This preliminary will sufficiently indicate my purpose, Mr. Editor, in offering you the following paper for publication.

Very generally there is a preliminary stage, marked by obvious symptoms. Less frequently the access is sudden, and not preceded by prodromic phenomena. Under the former condition of things, we may expect a more protracted but less severe case; while from the latter circumstance, more gravity in its symptoms and effects and shortness of duration are to be apprehended. The nervous system and digestive organs are the more prominent subjects of premonitory derangement. Lassitude, muscular debility, trembling of the extremities, uncertainty and unsteadiness of gait, easily induced fatigue, shortness of breath, and palpitation of the heart, often present in the precursory stage, make up that ensemble of symptoms known by the name of tires. Connected with these symptoms, we have loss of natural appetite, craving for ardent spirits, nausea, and, upon much exertion, vomiting, sense of oppression in the epigastric region, constipation, and that general but vague uneasiness which renders the patient restless and sleepless. Sometimes, however, he is dull, stupid, and indifferent to surrounding circumstances.

After the continuance of the above symptoms for a longer or shorter time, or in cases where they are not present, in consequence of the application of some exciting cause, the disease commences to manifest itself in a decided manner, when the following state of things may be observed: urgent thirst, vomiting every few moments, with distressing nausea between times, loathing of food, burning sensation and feeling of weight or load on the stomach, and obstinate costiveness, which, when we first see the patient, has probably lasted for several days. The substance ejected from the stomach may be the ingesta merely as water or food with a little mucous, but nearly always it is colored, light green, blue, indigo, or so very dark as to seem black. Upon examination of the ejected material, it will be found composed of two parts, which separate soon after it is discharged into a transparent liquid, nearly or quite colorless, and a more solid collection of coagula (probably the mucous secretion of the stomach) floating in it. The different color of the solid portion causes the various shades of color above mentioned. The taste of this ejected substance is sour, acrid, and sometimes, in the latter stages, otherwise offensive. It generally effervesces with the sup. carb. soda. Often, in the latter stages, it is so very dark as almost precisely to resemble black vomit. The alvine discharges which take place spontaneously, are very dry, scanty, and difficult to extrude, often assuming the shape of small, hardened balls, not generally altered much in color or other properties, except in respect of moisture. The abdominal parieties are retracted and hard, and the pulsations of the abdominal aorta are visible, and perceptible to the touch from the epigastrium to its division. This phenomenon, no doubt, results from the dryness of the fæces and lack of fluid and gas in the bowels, thus rendering their contents better conductors of motion. The surface is generally dry, but not more commonly elevated in temperature, and is usually of a dingy or dirty color. The tongue is almost always coated with a white, brown or yellow layer. The pulse, in the majority of cases, early in the disease, is not much or remarkably altered, perhaps not at all. The breathing is generally more or less embarrassed by a sense of oppression or weight on the chest, interrupted and sighing. This oppression is often a prominent and distressing symptom. A peculiar fetor emanates from the lungs and mouth, which may be regarded as characteristic, and, when once observed, will be readily recognized by even a superficial observer. This smell is so remarkable, and sometimes so strong, as to be perceptible anywhere in the room, adjoining rooms, or even several yards from the house. Experienced practitioners might, in many instances, form their diagnosis before entering the house, from the peculiar odor on the premises. It is not uncommon, in bad cases, for delicate and sensitive persons to be so much affected by this oppressive and disagreeable smell, as to render them incapable of remaining at the bedside, or in the room of the patient. Its almost corrosive pungency would convince an unbiased observer, through one of the senses at least, that there was a peculiarity in it. Comparison being one of the best means of acquainting a person with an unfamiliar subject, I have sought and found one which I think will be regarded as appropriate.

It resembles, almost exactly, a strong odor of chloroform, mingled with the smell of animal secretions. Say a smell of chloroform and the breath of a patient salivated, and we would have it almost precisely. The kidneys secrete scantily, and the urine, though not altered in color or appearance, emits the same odor of the breath. The spirits are dejected, and the patient is despondent. Should no relief be afforded, and the disease continue to advance, the respiration is more embarrassed, and accompanied by a sense of sinking, or as of a void which the patient is unable to fill by the most strenuous efforts at inhalation. The irritability of the stomach increases. The ejected matter, if not dark before, now becomes very dark and scanty, having the appearance of coffee-grounds, and is thrown about without any regard to cleanliness, and with very little effort, the patient either being listless and apathetic, or employed absorbingly with the respiratory efforts. The surface becomes cold, the pulse is small, frequent, and finally fails, which often occurs several hours before death. Not unfrequently, some time before death, while the pulse is small, frequent and weak, the respiration is slow and irregular, to such a degree as to lead the bystander to believe that it is entirely suspended ; coma supervenes, which gradually deepens into death. Again, in some cases, after the lapse of several days or a week from the commencement, the bowels either act spontaneously, or having been moved by cathartic medicine, become swollen, tympanitic, irritable, and painful, the discharges are watery, with mucous floculi floating in them, tinged with blood, or scybalous and fœtid. The tongue is dry, red and chapped, or coated with a dark, dry fur, teeth covered with sordes, the fauces dry, red, stiff and sore, deglutition difficult, regurgitating emesis of the dark fluid above described, dorsal decubitus, with knees drawn up, retained urine, (often,) coma, small, frequent and weak pulse, hot abdomen, and cool or cold extremities. The inferior maxillary falls, the eyes become suffused with mucous and tears, and injected, and death, after a period varying from several hours to several days, gradually steals upon the patient. If, however, the case is to terminate favorably, after a few copious alvine discharges, the burning sensation at the stomach with the nausea becomes less, the vomiting gradually subsides, muscular debility becomes less, the tongue cleans off, the patient experiences a returning but capricious appetite, desiring apparently the most inappropriate diet. The smell of the room is more tolerable, and a protracted convalescence ensues, attended, unless carefully guarded, with many of the symptoms detailed as sometimes promonitory, and not unfrequently their chronic continuance encourages the popular opinion that it never can be entirely eradicated from the system. Occasionally, though not usually, in slight cases, the convalescence is more rapid and complete in a few days, leaving the patient in the enjoyment of good health.

The above is a description of what may be called the simple variety, in contradistinction to two other forms which it sometimes assumes. It is by far the most common met with in this vicinity at the present time. The two varieties alluded to may be termed, according to the nomenclature in vogue, congestive and inflammatory, according as the attending phenomena indicate the presence of either of these general conditions of the system. The congestive variety is very rapid in its progress, and among the most fatal diseases, equalling, in mortality, (according to the number of cases,) the most fearful varieties of congestive miasmatic fevers of the same regions. This is probably the character which the disease more commonly assumed in the earlier periods of this country's history, as it was then considered the most surely fatal of diseases, in which death usually ensued in a very few days. After suffering from the premonitory symptoms some time, or, otherwise, violent vomiting and incessant retching, a sense of impending suffocation, extreme anxiety, insatiable thirst for water, and often for alcoholic liquors, coldness of the extremities, a profuse, watery perspiration, small, weak and frequent pulse, a glassy appearance of the eyes, great prostration of strength, restlessness, jactitation, suppressed urine, and a wandering condition of the intellect, soon convince us of the great danger of our patient. And the peculiar smell, the condition and appearance of the abdomen and bowels, the almost insatiable thirst for alcohol, and other characteristic symptoms, plainly indicate to the experienced the true nature of the disease. Often this catalogue of symptoms continues without much change, except in intensity, for from twelve hours to two or three days, when, if relief is not afforded, death closes the scene. In other instances, and they are not unfrequent, coma supervenes upon these symptoms, or without much coldness or change in the pulse, and continues to the end. This form of the disease has never, in my experience, presented itself in any other class of patients than the enfeebled or old and decrepid, or supervened upon the simple form; but I am informed, by reliable and intelligent practitioners, that it is not so restricted where the poison is more violent and concentrated—that even the most robust succumb to this array of symptoms in a very short time.

The inflammatory is midway, in respect to fatality, between the two already described, and although more frequent than the congestive, is fortunately not so common as the simple. The distinctive symptoms are, high arterial excitement, severe headache, heat of the surface, flushed face, sometimes delirium of an active character. Often there is tenderness on pressure over the stomach and bowels. The pulse is full, hard, and somewhat quickened, ranging as high as 100 to 110 in the minute. Otherwise, the symptoms do not differ from the simple kind. Here we have much reason to fear the existence or supervention of active inflammation in the stomach and bowels. Besides these forms, it may be complicated with any of the endemic or epidemic diseases of the country in which it prevails, and not unfrequently in the debility consequent to or attendant upon the progress of these maladies, it finds an efficient exciting cause.

It would be obviously unnecessary to draw a differential diagnosis in which to pass in review all the diseases which in the least resemble it. The flatness and pulsation of the abdomen, obstinate costiveness, dry condition of the fæces, vomiting the peculiar substance described, oppressed respiration, and, above all, the unmistakable and unique odor of the breath and everything about the room of the patient—perhaps I might add, also, the often unbecoming and indelicate yet urgent thirst for ardent spirits—form a picture that is hard to match by either any whole or parcels of disease. It is a matter of no small difficulty to speak with accuracy of the anatomical appearances of this disease, as the prejudices of the people in new countries prohibit, in most instances, (where it is most prevalent,) post mortem examinations. Dr. Trafton, who has examined several cases, says that he has found superficial inflammation of the mucous membrane of the stomach, with a rigid contraction, amounting to closure of the pyloric orifice, a dry condition of the fæcal contents of the bowels, and almost complete absence of gas. The fæces are generally so dry that they are unusually hard, and seem to contain no fluid. No appearances of disease elsewhere, worthy of note, were observed. In examining animals that die of this disease, the half-digested food in the stomach is found formed into hard, round balls, often, by the peristalic action of that organ, of various sizes; and the fæces in the large bowels are so dry as that they readily crumble into powder upon the application of slight force. Some observers have found—in the congestive form, I suppose—obvious injection of the membranes and substance of the spinal cord, brain, and sometimes effusion of serum, and even blood. These appearances readily account for the additional symptoms presented in this variety of cases. The inflammatory form we would expect to present evidences of inflammation in the different viscera to which the symptoms pointed during life. But I have no reports from which I can adduce demonstration. Instances, no doubt, occur in the s mple variety, after a protracted duration, which would exhibit post mortem appearances of treatment as well as the disease. And I make this observation, not as a censure or criticism upon any course of management; for in no other disease, so frequently encountered and cured in this country, is there so little harmony among medical men, as to the nature of the disease or its proper curative requisites. And here I may venture the remark, that, often, enough allowance is not made in examining subjects, dead of idiopathic diseases in general, for the local appearances induced by extraneous agents. Probably, too, disputes have arisen among eminent medical men as to their nature, from a want of such considerations. The prognosis of simple cases, if properly treated, may be almost always favorable, and they often get well without much treatment, and occasionally in spite of improper management. A strong asthenic tendency or inflammation of the bowels, manifested by tympanites and soreness, with mucous and fœtid dejections, augur badly. In the congestive form, the patient is imminently dangerous, and our prognosis should always be unfavorable, owing to the degree of congestion. But the worst cases may often be cured by active and appropriate interference early in the disease.

In approaching the etiology of milk-sickness, I am aware that I encounter a perplexing question. Hypothetical facts, in countless numbers of causative agencies, have been presented, yet all are unsatisfactory. Vegetable, mineral, and miasmatic etiological phantoms have been marshalled to support the favorite hypothesis of different speculators, and have vanished before scrutiny as suddenly as their legendary representatives before the light of day. But much that is profitable of the circumstances under which it is observed to prevail, is known. Certain localities are known to be more prolific of its propagating poison than others. Their physical characteristics are not peculiar. It is in hills, valleys and ravines, on bottom land and the "flats," as they are called. Its localities may be clothed with timber, underbrush and herbage of every description common in the West and South. To give the northern, southern, eastern or western boundary of the district in which it prevails and has prevailed, would be impossible with the data in my possession. The virgin soil, in its own contents or spontaneous productions, is the fountain of the poison, and, with slight exceptions, perhaps the whole West and South-West contain sections of greater or less extent which have produced the disease during some time in their redemption from their forest condition. The autumnal period of the year is by far the most productive, although it may occur, in a more limited degree, during any season of the year. For the most part, like miasmatic fever, it disappears upon the accession of severe frosts. Its prevalence is favored by dry weather, a dry fall being much more favorable to it than any other. That man is sometimes the original recipient of the poison, there can be many cases brought forward to prove, without violating the latitude of propriety generally given in observations of the kind. It is freely admitted, at the same time, that a large majority of patients receive it from poisoned animal food, as beef, mutton, butter, &c. Indeed, it has been so common, and the people are so well aware that milk may impart the disease, that it has been named milk-sickness by almost universal consent. Whatever this poison may be, some of its effects resemble very closely the phenomena produced by the recognized animal poisons, in some respects at least. One of these is, that it undergoes multiplication in the system. From a small amount received, it may be through the stomach or lungs, it so far increases as to make every part of the system through which it circulates so virulently poisonous that very small portions eaten set up the same action in another animal, and so the process probably might be carried on *ad infinitum.* The calf that takes it from the milk of its mother, by its flesh may impart it to man. Or, as is frequently observed to be the case, a dog may receive it from the calf and impart it to the buzzard that eats his flesh, and the latter, unable to rise, dies on the ground. The skinning of cattle dead from this disease, often imparts to the person performing this operation, by the effluvia emanating from the exposed body, the most virulent form of the disease. Is there any vegetable or mineral poison which, after having gained admittance into the system in sufficient quantities to produce death in the victim, imparts so much of its own substance to a small handful of the flesh, or insinuates itself so universally and copiously into every drop of the fluids, as to cause death by the ingestion of the former or the inhalation of a small snuff of the latter, while emanating in imperceptible vapor from the denuded animal, to awaken a destructive action in a healthy one similar to the disease of which it had died? If so, what can it be? Certainly, nothing with the modus operandi of which we are acquainted. The length of time that the poison remains in the system, without starting the train of morbid action, will vary, from several circumstances, viz: the intensity of its power, or the quantity of it; the capacity of the system to resist disease; and absence of any exciting cause. When sufficiently intense, there is no doubt that it immediately overcomes the power of resistance of the system, and the disease is awakened suddenly into existence. This is more likely to be the case in persons previously debilitated by other diseases, exposure, or scanty living. Sometimes, however, such is the case with the robust. More frequently, a considerable time elapses before it breaks forth actively. Lactation seems to exercise a protective power, probably by eliminating the poison through the secretion. Quietude of body and of mind is also favorable to resistance. One of the most effectual exciting causes is fatigue produced by exercise to lassitude. This is so well understood by the people, that they often test the soundness of their animals by chasing them until they are fatigued, when, if the poison is lurking in their system, they will easily tire, tremble, and fall, or lie down. Fatiguing exercise produces the same effect upon man. Excesses in the use of ardent spirits is a powerful source of evil in individuals exposed to the action of the poison. Gluttony, or eating diet of difficult digestion, the debilitating passions, protracted exposure to the heat of the sun, awaken the disease in persons predisposed.

What is the nature of this disease? Some of the learned of our profession doubt the existence of it as a distinct affection, and merge it into some of the already recognized and described maladies. Some describe it as gastritis; some cerebro-spinal meningites; others regard it as bilious vomiting, or miasmatic fever, &c. It is a matter of astonishment to all who are practically acquainted with it, that their opinion should be questioned, their intelligence and judgment called to account, when they pronounce it a disease *sui generis*, and that, too, not upon correct theoretical grounds, but upon the presumption of closet critics, who have never seen it. It is also as astonishing as it is detrimental to the interests of the medical profession, particularly in the West, that a good and reliable section on this disease cannot be found in any of the deservedly popular text books. Indeed, the young practitioner, here and elsewhere, where it prevails, must learn all he knows of it from his neighbors—from the people themselves; and this, too, upon a very frequent disease. It is to be hoped that the prejudices of authors will not long thus deprive us of so valuable a part of their books, if they desire them to be read and approved by a large and respectable portion of the profession. It is regarded as a real, *bona fide* disease, and not a creation of the brain, by a large majority of the physicians in whose midst it prevails, and it is unfair not to listen to their testimony, and, if possible, supply their wants. I think I represent the general opinion of medical men of intelligence where it prevails, in saying it is a blood disease—that it pervades, through this fluid, the whole system, and that local inflammations and congestions are not essential accompaniments, and occur, as the effects of the long continuance of irritation, as of the stomach or the separate but contemporaneous action of their own causes. Is there any disease but a blood disease that emits a peculiar odor? We talk of the smell of typhus and small-pox, but we do not think of experiencing any peculiar odor in pneumonia or gastritis. This distinction is so well understood, that we expect an idiopathic fever instead of inflammation, when we detect an unusual smell about the patient which cannot be accounted for in any other way than by exhalation from his person. Individuals, who have paid any attention to the smell of blood in a dissecting room, will readily discover a difference in the odor of cholera and fever patients, from those who die of inflammations.

With respect to treatment, experience proves that in the simple form the all-important indication is to open the bowels and keep them in a soluble condition throughout the management of the case. Upon the early fulfilment of this indication, success depends. This irritability of the stomach is a seriously embarrassing circumstance, and should, as far as possible, be removed. Too much time and attention must not be devoted, however, to this symptom, for at first our attempt will generally prove abortive, and we should remember that it ceases, for the most part, spontaneously, so soon as free alvine evacuations are procured. The most that is deemed advisable in the commencement, is the application of sinapisms to the epigastrum, and the administration of pellets of ice to allay the burning sensation of the stomach. We should not hesitate to begin, without waiting for any decided effect from remedies to allay vomiting, the administration of, and frequently repeat, purgatives until they operate. A Seidlitz powder, with a double proportion of salts, every hour, is an agreeable as well as effectual agent for this purpose. Or, one drachm each of cream tart. and white sugar, in two ounces of infusion of Senna, given as frequently. Compound powder of jalap may also be used in teaspoonful doses every hour, in syrup. Rochelle and epsom salts, &c., are quite available in many instances.

Probably a part of each dose of any of these medicines will be retained, although vomiting continues. The quiescent condition of the stomach, immediately succeeding the act of vomiting, is the best time to give the medicine. By persevering in giving the medicine, notwithstanding the rejection of the most of it, changing from one of the above mentioned articles to another as the patient becomes tired of any one of them, we may, generally, in the course of twenty-four hours—sometimes sooner, sometimes a little later—procure free purgation of fluid fæces, when our patient will be much relieved. Conjointly with the administration of medicines per orem, we may introduce them per anum. After we have made some peristaltic impression on the bowels, and not before, I think, will it be worth while to trouble the patient with injections. Judging from my own experience, we need not commence them in less than twelve or eighteen hours after we begin with the cathartics. Much the most effectual way of using them is to pump through a tube a large quantity of warm water high up in the colon; say from a half to a gallon at a time, with epsom salts dissolved in it, or oil floating on it. One of these injections, well-timed, will shorten the duration of suffering many hours, in the most of cases. It will be seen that the purgatives recommended are hydrogogues, such as induce serious infusion from the intestinal tube. This, I think, is a subject of no small importance, as they operate with more facility, and relieve by furnishing the fluid in which to suspend the dry, hardened fæces, and thus provide for their easy expulsion.

Many practitioners rely on the salts of opium to allay the irritation of the stomach before they commence the purgative plan. But, although I have used them, I do not now—and, when I did, have in every instance regretted having done so—from the fact that they have failed to have the effect, in the most of cases, and always rendered the bowels difficult to move. Yielding to the appetite for strong drink, some practitioners—among whom I may mention my friend Dr. DeBruler, for whose opinion I have the greatest respect—allow whisky, wine, or brandy, *ad libitum*, at the same time they give the saline cathartics; epsom salts and whisky being, as they say, all that are necessary to the cure. Others rely upon large and frequently repeated doses of calomel, because less easily rejected from the stomach, while it effectually operates upon the bowels.

But the bad effects of a protracted salivation, which I have seen follow this plan, and sometimes even worse effects, have deterred me from using this mineral with such freedom. An ingenious and (according to Dr. Bacon, its originator) highly efficacious method of treating the early stages of this disease, is to administer large boluses of blue mass—so large that they cannot be ejected on account of the weight and size. One every half hour or every hour until free discharges are procured. And if there is more than ordinary delay in their action, the Doctor punches a hole in the centre of the boluses, introduces a drop of croton oil. Dr. B. tells me he does not fail to get free evacuations in less than twenty-four hours by this plan. Antacids, though apparently indicated, are of little use until after evacuations have been produced, when they are grateful, and help relieve the burning in the stomach. In the subsequent management, the bowels he kept free by medicines which produce thick and consistent operations. A pill, of equal parts of rhubarb, aloes, and capsicum, is one that answers the purpose very well. If the liver should fail to act with the bowels, a little blue mass is all that I have found necessary. It is often the case that the stomach does not regain its vigor for some time, and will need some aid from the bitter infusions, quinine or some of the ferruginous preparations. Ordinarily, the above treatment will be sufficient to conduct the patient through a simple attack to a speedy recovery. But, in grave cases, and where they have not been treated early, it is often otherwise. When the bowels begin to move, although the stomach is somewhat relieved, the distress in a great measure continues, there is tenderness on pressure over the epigastric region, and inflammation as the result of intense or protracted irritation. Under these circumstances, mucilaginous drinks, cooled with ice-water, and a blister over the stomach, will assist very much in the case. It is always best in these cases, as far as practicable, to keep the bowels open by injections, instead of using cathartics by the mouth. In another set of cases there is great debility, indicating the use of stimulants. The carb. of ammonia will often give great relief; but egg-nog, brandy, and sul. ether often become necessary, and should be freely used with animal broth, &c. We may support the circulation, too, by blisters to the extremities, when these are cold. In the inflammatory form, while attending to the treatment essential to the simple form, it will also be necessary to take blood to an extent sufficient to subdue arterial reaction or inflammatory complications, being guided by the general principles governing this condition in other circumstances, strong pulse, heat of the surface, &c. Much activity and energy and treatment are the only conditions of success in the congestive form.

Injections into the bowels, of warm water, and, in the more algid and prostrate cases, brandy and turpentine should be added. To injections into the colon of the size and character above mentioned, put two ounces of the oil of turpentine and the same quantity of brandy. At the same time, friction of hot oil of turpentine to the extremities, or large sinapisms, if the coldness and prostration be great, may be applied so as almost to cover up the legs and arms with them. If these are not at hand, or in conjunction with them, hot bottles of water, the steam bath, hot bricks, or irons, applied all around the limbs and body of the patient, will aid materially in giving energy to the almost overwhelmed functions. Internally, it will be well to make warm whisky or brandy toddy the vehicle for the administration f the purgative medicines so necessary in the treatment. Should we succeed in bringing about re-action, and reducing, by external and internal stimulants, the case to either the simple or inflammatory form, the treatment must be conducted in the manner described above, It is not uncommon, in cases of this kind, for the head, chest and abdomen all to be preternaturally warm, while the extremities are cold. In such, internal stimulants are not well borne. The external, however, may be used as above directed, while cold water and ice will advantageously succor us, when applied to the trunk and head. As palliatives during the progress of disease, soda, lime water, chalk, &c., when they can be used without interfering with the more necessrry remedies, will be very agreeable. Yeast is also said to be very grateful to the patient, and useful in allaying vomiting. This was a favorite remedy with the late Dr. Trafton, of this place, who attributed effective curative virtues to it. It is but a just tribute to the memory of the late Dr. Wm. Trafton, to say that more is due to him for the successful management of mik-sickness, than any other man in this part of our State.—*Nashville Journal of Medicine.*

